# Lymphatic and vascular anatomy define surgical principles for radical treatment of distal duodenal and proximal jejunal tumors

**DOI:** 10.1007/s00464-025-11909-9

**Published:** 2025-07-07

**Authors:** Teodor Vasic, Milena Stimec, Bojan Vladimir Stimec, Bjørn Edwin, Dejan Ignjatovic

**Affiliations:** 1 Clinic for Digestive Surgery, Faculty of Medicine, University Clinical Centre of Serbia, University of Belgrade, Belgrade, Serbia; 2 Anatomy Unit, Faculty of Medicine, University of Geneva, Geneva, Switzerland; 3 Intervention Centre, Oslo University Hospital, Oslo, Norway; 4 Department of Digestive Surgery, Akershus University Hospital, Oslo, Norway; 5 Institute of Clinical Medicine, Faculty of Medicine, University of Oslo, Oslo, Norway

**Keywords:** Jejunum, Duodenum, D3 volume, Jejunal artery, Lymphadenectomy, Surgical technique

## Abstract

**Background:**

The arterial ligation level and the lymphadenectomy extent for surgical treatment of distal duodenal/proximal jejunal tumors are not standardized.

**Aim:**

To define morphometric and topographic specifics of the superior jejunal artery (SJA) and the superior jejunal vein (SJV), and the width of arterial lymphovascular bundles through lymphatic clearances. By extrapolating results from two methodologies, the goal is to determine the arterial ligation level and the lymphadenectomy extent for duodenojejunal tumor treatment.

**Material and Methods:**

The first series included an analysis of preoperative 3D-CT vascular reconstructions from 97 patients. The second series included 11 dissected cadavers where the course of the proximal mesenteric lymphatics was followed. The SJA was defined as the uppermost jejunal artery (JA) counted from the ileocolic artery (ICA) origin.

**Results:**

SJA proper was present in 72 cases (74.2%). The mean SJA caliber was 3.4 ± 1.2 mm. SJA originated 17.5 ± 16.8 mm cranial to the middle colic artery (MCA) origin and was found cranial to it in 84 cases. SJA originated caudal to the inferior pancreatic border in 80 cases (82.8%) and cranial in 17 (17.2%). SJV coursed anteriorly to the superior mesenteric artery in 29 cases (29.9%). The distances of the cranial and caudal lymphatics following SJA at the level of the arterial origin were 1.15 ± 0.53 mm and 0.6 ± 0.35 mm.

**Conclusions:**

To achieve adequate lymphatic clearance, it is crucial to ligate the tumor-feeding SJA at its origin. The conical arrangement of the SJA lymphatics underscores the necessity of a mesenterectomy extending both cranially and caudally, including lymphadenectomy along the adjacent JA to the level of the first arcade.

**Trial registration:**

“Surgery with Extended (D3) Mesenterectomy for Small Bowel Tumors” registered at https://classic.clinicaltrials.gov/ct2/show/NCT05670574 NCT05670574

**Supplementary Information:**

The online version contains supplementary material available at 10.1007/s00464-025-11909-9.

## Introduction

Gray’s Anatomy states that the distal duodenum and proximal jejunum are supplied by the superior jejunal artery (SJA) and the inferior pancreaticoduodenal artery (IPDA), which may share a common trunk [1]. In the early twentieth century, anatomists observed that small bowel (SB) lymphatics are three to four times more abundant than blood vessels. They discovered that duodenojejunal lymph vessels emerge from the bowel wall, penetrate the upper mesentery, and run parallel to the proximal jejunal arteries (JAs), forming lymphovascular bundles that drain into the main lymph nodes (LNs) that lie along the superior mesenteric artery (SMA). Moreover, lymph vessels can course directly to the main LNs, bypassing LN stations in between and interconnecting along the way [1–3].

Due to the complex lymphovascular anatomy and low incidence, surgical management of distal duodenal and proximal jejunal tumors is challenging. The guidelines for localized SB tumors recommend segmental surgical resection with en bloc removal of at least eight LNs [4, 5]. The extent of lymphadenectomy varies, and several surgical techniques for treating duodenojejunal tumors have been reported, including radical duodenopancreatectomy, segmental SB resections, and full-thickness wall excisions [6–8]. Recent studies highlight the importance of LN dissection for duodenojejunal tumors [9]. Pooling the data from the SEER database, Tran et al. demonstrated that the higher number of harvested LNs in 1387 patients directly correlates with greater median survival for duodenal adenocarcinomas (1–5 LNs: 44 months; 6–10 LNs: 82 months; > 10 LNs: 99 months; *P* = 0.008) [10]. Additionally, Cloyd et al. reported an average of 6.8 ± 7.8 LNs harvested (positive LNs 1.6 ± 2.7) for segmental distal duodenal resections [11].

The primary objectives of complete mesocolic excision (CME), central vascular ligation (CVL), and D3 lymphadenectomy in colorectal surgery are to enhance LN harvest and achieve intact removal of lymphovascular bundles. This approach aims to prevent spillage of cancer cells by including the adequate lymphatic clearances [12, 13]. Surgery for distal duodenal and proximal jejunal tumors has not achieved the standardization of surgery for colorectal cancer [4].

This study aims to analyze the morphometric and topographic specifics of the SJA and the superior jejunal vein (SJV), as well as the width of arterial lymphovascular bundles defined through lymphatic clearances. By extrapolating the results from two distinct methodologies, it aims to define the level of arterial ligation and the extent of lymphadenectomy for a radical surgical technique in the treatment of distal duodenal and proximal jejunal tumors.

## Material and methods

In this cohort study, we have analyzed the anatomical relations in the upper mesentery in both a clinical and a post-mortem series of subjects.

The first series comprised data from a 3D-CT mesenteric vascular anatomy reconstructions dataset involving 97 patients enrolled in the “Surgery with Extended Mesenterectomy for SB Tumors” clinical trial. This trial received approval from the Regional Ethical Committee South-East, Norway, and was registered on clinicaltrials.gov [14]*.* Before surgery, each patient provided informed consent. The inclusion criteria were age above 18 years, radiologically or scintigraphically diagnosed SB distal duodenal and proximal jejunal tumors, and ability to tolerate general anesthesia. As part of the trial, the personalized mesenteric vascular anatomy of the area was reconstructed in 3D, based on the preoperative staging CT dataset for each patient. All patients underwent D3 volume lymphadenectomy as defined by Spasojevic et al. [15].

The second series consisted of 11 embalmed human bodies from the body donor program of the Anatomy Unit, Faculty of Medicine, University of Geneva, Switzerland. The use of human cadaveric material did not require an Institutional Review Board (IRB) decision, as it was done following the Federal Act on Research involving Human Beings (Human Research Act, HRA), Guidelines of the Swiss Academy of Medical Sciences, and the principles of the Swiss Society of Anatomy, Histology, and Embryology [16–18]. Donors formally consented to the use of their body parts for research purposes after death by signing a body donation statement form [19].

### Preoperative CT reconstruction

Each patient from the first dataset underwent standard preoperative investigation for SB tumors, as per Norwegian guidelines. This involved an abdominal and pelvic CT scan, which was used for 3D vascular reconstruction. No new scans were ordered. The CT dataset was analyzed using a 2D multiplanar reconstruction with a maximum intensity projection and a 3D volume-rendering technique using the FDA-approved OSIRIX MD v.14.1.1 64-bit image-processing application software (Pixmeo, Bernex, Switzerland). The root of the mesentery and adjacent structures were precisely depicted using manual segmentation via the serial application of Region of Interest (ROI) through editing tools: Open polygon, Pencil, and Repulsor. After attributing pixel values outside ROIs to air and inside to the original value, the virtual 3D model was obtained by volume rendering (VR). The identification, internal caliber, and course of the vascular structures within the predefined D3 volume were analyzed according to the postulates for CT angiography of mesenteric vessels [20].

### D3 volume

For this study, the D3 volume is defined by the following borders, according to Spasojevic et al. [15]: cranial border: 5 mm cranial from the line that passes through the origins of the MCA and the gastrocolic trunk of Henle (GTH). When the SJA serves as the tumor-feeding artery, the D3 volume extends 5 mm cranial to it; lateral border: 1 cm to the right of the line along the right side of the SMV; caudal border: 1 cm distal to the line that runs through the origins of the ICV and the ICA; medial border: the line along the left side of the SMA; Anterior surface: the peritoneum; posterior surface: the mesofascial plane.

### 3D vascular reconstruction data collection

The 3D-reconstructed images and written reports were used for data collection. The JAs were counted from the ileocolic artery origin (ICA) cranially, following the definition provided by VanDamme and Bonte [21]. Thus, our study group defined the farthest JA from the ICA, commonly known as the first JA, as the SJA. The origin of the SJA was identified and described, noting whether it originated directly from the SMA (SJA proper) or if there was a common trunk with the IPDA. The level of origin’s relation to the inferior pancreatic border and splenic vein (SV) was registered (i.e., originating cranial or caudal to the lower pancreatic margin). The caliber of the SJA was measured, as well as the distance between the SJA origin and origins of the ICA, middle colic artery (MCA) and adjacent JA (the first caudal to the SJA). The number of JAs was reported*.* The presence of any JA cranial or caudal to the origin of the MCA was registered.

The SJV was identified as the uppermost vein counted from the ileocolic vein (ICV) and its confluence. The relationship to the SMA was described as posterior or anterior. The path of the inferior mesenteric vein (IMV) was registered as it was noted whether it drained into the superior mesenteric vein (SMV), the SV, SMV–SV confluence or the JV trunk. In addition, the position of the IMV related to the SMA and MCA origin was noted. We observed the eventual existence of the left SMV (pseudo-bifid SMV), which our study group previously defined [22].

### Anatomical dissection

The exclusion criteria for this study were chronic or acute diseases of the digestive tract, past abdominal surgery, and infectious diseases, but the age was not limited. The bodies were injected with a Jores solution, consisting of formaldehyde, chloral hydrate, Carlsbad salt, and distilled water. After that, the cadavers were placed in vacuumed plastic bags and kept in the refrigerator at 5 degrees centigrade. After conditioning to the ambient temperature, the minute dissection (performed by BVS and MS) began with a wide semicircular incision of the anterolateral abdominal wall, enabling access to the infracolic compartment of the peritoneal cavity. The greater omentum and the transverse colon were pulled cranially, and the SB mesentery was spread out from the duodenojejunal junction onward. The anterior leaf of the mesentery was carefully removed from the wider area over the superior mesenteric vessels, using the ICA and MCA folds as landmarks. The subserosal fatty tissue was removed by gentle scraping using narrow medical spatulas, micro-dissection scissors, small tweezers, and curved forceps. The next step was to follow the arborization of the SMA branches and the SMV affluents, preserving the accompanying and neighboring lymph vessels, their network, and the lymph nodes. This procedure necessitated the use of the 5 × magnifying glass with a fluorescent ring lamp. The course of the lymph vessels was followed, and they were separated from the underlying nerve plexuses and connective tissue without disturbing their native anatomical position. Throughout the dissection, the working field was regularly sprayed, i.e., kept humid by a phenol solution, preserving the original consistency. Once the proximal jejunal lymph and blood vessel network was highlighted, the dissection continued in the same manner along the MCA course, along the left colic angle, and on the posterior aspect of the duodenojejunal junction, using the Treitz suspensory ligament as a pivot. The syntopy of the anatomical entities in the area was carefully observed, and then the dissection was continued along the SMA upstream to its origin on the abdominal aorta. The following distances were measured with the aid of the digital caliper (S Cal Work, Sylvac, CH):

- Between the origins of the SMA and the SJA
- Between the SJA and the neighboring accompanying lymph vessels, at origin and at 1 cm distal to the origin (cranial and caudal lymphatic clearance)

Also, the following relations were noted: the origin of the SJA and the inferior pancreatic border, the existence of proximal JVs if the IMV opened into the SMV, and the possible common trunk for the IPDA and the SJA.

## Statistical analysis

Observed characteristics were expressed as mean values ± standard deviation and the median number with interquartile range (IQR). We used the Kolmogorov–Smirnov test to assume normality. IBM SPSS Statistics 21 (Chicago, Illinois, USA) was used for the analysis. All metric units were measured in millimeters.

## Results

A total of 108 subjects were included in this study: eleven cadavers in the post-mortem investigation at the University of Geneva and 97 cases of preoperative 3D-CT reconstructions from the clinical trial at Akershus University Hospital (60 males; median age 62.5 years, IQR 18).

### Preoperative 3D vascular reconstruction series

#### The arteries

The SJA proper was present in 72 cases (74.2%), while the remaining 25 cases shared a common trunk with the IPDA (25.8%). In 84 cases (86.6%), the SJA originated cranial to the MCA origin, while in 13 cases, it originated caudal to the MCA origin (13.4%). The SJA was found to originate cranial to the inferior pancreatic border and caudal to the SV in 17 cases (17.5%). The mean ICA-MCA distance was 25.6 ± 11.3 mm. The ICA-SJA distance was 44.1 ± 13.8 mm, and the SJA-adjacent JA distance was 12.0 + 7.0 mm. The mean SJA caliber was 3.4 ± 1.2 mm. The median number of JAs was 4 (2–7).

#### The veins

The median number of JVs was 2. The single JV trunk exclusively drained the jejunum in 36 cases (37.1%), and in 22 cases, the caliber exceeded 10 mm. The mean caliber of SJV was 9.4 ± 5.1 mm. The mean ICV–SJV distance was 31.7 ± 13.6 mm. The SJV coursed anterior to the SMA in 29 cases (29.9%), while the posterior course was present in 68 cases (70.1%). The IMV drained into the SMV in 45 cases (46.4%), SMV–SV junction in 6 (6.2%), SV in 42 (43.3%), and JV trunk in 4 (4.1%). In 55 cases (56.7%), the IMV crossed the SMA anteriorly, 28.1 ± 12.4 mm cranial to the MCA origin. When the IMV drained to the SV, it coursed left to the SMA. In cases when the IMV drained into the JV trunk, the latter was noted as the left SMV (pseudo-bifid SMV). [22] The left SMV crossed the SMA anteriorly in all cases.

### Anatomical dissection series

Of the eleven specimens included in the post-mortem studies, there were six females and five males, aged 42–95 years. The SJA came off the left lateral aspect of the SMA in all eleven cases. The origin of the SJA was found to be caudal to the inferior pancreatic border in eight patients out of the eleven (72.7%). That is, in only three patients, the artery originated cranial to the inferior pancreatic border. In one patient (9.1%), the inflow of the SJV to the SMV was cranial to the level of the SJA origin and in one patient (9.1%), the SJV opened into the IMV. In the remaining nine patients (81.8%), the SJA was more cranial than its homonymous vein. In none of these eleven specimens did the SJA and the IPDA come off the SMA as a common trunk. The mean measured distance from the aortic origin of the superior mesenteric artery to the SJA was 42.3 mm (29.7–61.5 mm). The complete dissection dataset morphometrics are presented in Table 1.

**Table 1:** Morphometric analysis of the SJA and its lymphatic clearances obtained through the anatomical dissection of eleven embalmed human bodies

Subject sex	Age	SMA–SJA	CLyC	CLyC_1_	CdLyC	CdLyC_1_	SJA:IPB	IPDA–SJA cTr
M	73	32.9	0.5	7.6	0.3	2.35	Cr	N
F	82	33.85	1.05	5.7	0.2	3.3	Cd	Y
M	87	n/a	1.55	9.95	1.05	3	Cd	Y
F	94	50.35	1.1	5.3	0.4	1.25	Cd	N
F	90	46.6	0.7	5.5	0.3	1.55	Cd	N
M	42	50.1	0.5	5.25	0.35	1.3	Cr	N
F	92	29.7	1.6	10.35	0.9	4.05	Cd	N
M	85	30.4	1.75	4.5	0.8	1.65	Cr	N
M	95	46.25	0.7	3.85	0.4	1.05	Cd	N
F	78	41	2.05	8.8	1.25	4.75	Cd	N
F	82	61.5	1.3	4.95	0.6	3.1	Cr	N

All metric units are in millimeters

*M* male; *F* female; *SMA–SJA* distance from the SMA origin to the SJA origin; *CrLyC* lymphatic clearance between the lymph vessel (LyVe) and the origin of the SJA; *CrLyC*_*1*_ lymphatic clearance between the cranial LyVe and the SJA 1 cm distal from the artery origin; *CdLyC* lymphatic clearance (distance) between the caudal LyVe and the origin of the SJA; *CdLyC*_*1*_ lymphatic clearance (distance) between the caudal LyVe and the SJA 1 cm distal from the artery origin; *SJA:IPB* SJA origin position in relation to the inferior pancreatic border (IPB); *Cr* cranial; *Cd* caudal; *SJA* superior jejunal artery; *SMA* superior mesenteric artery; *SJV* superior jejunal vein; *IPDA* inferior pancreaticoduodenal artery; *Ly* lymph vessel; *Y* yes; *N* no

**Fig. 1:**
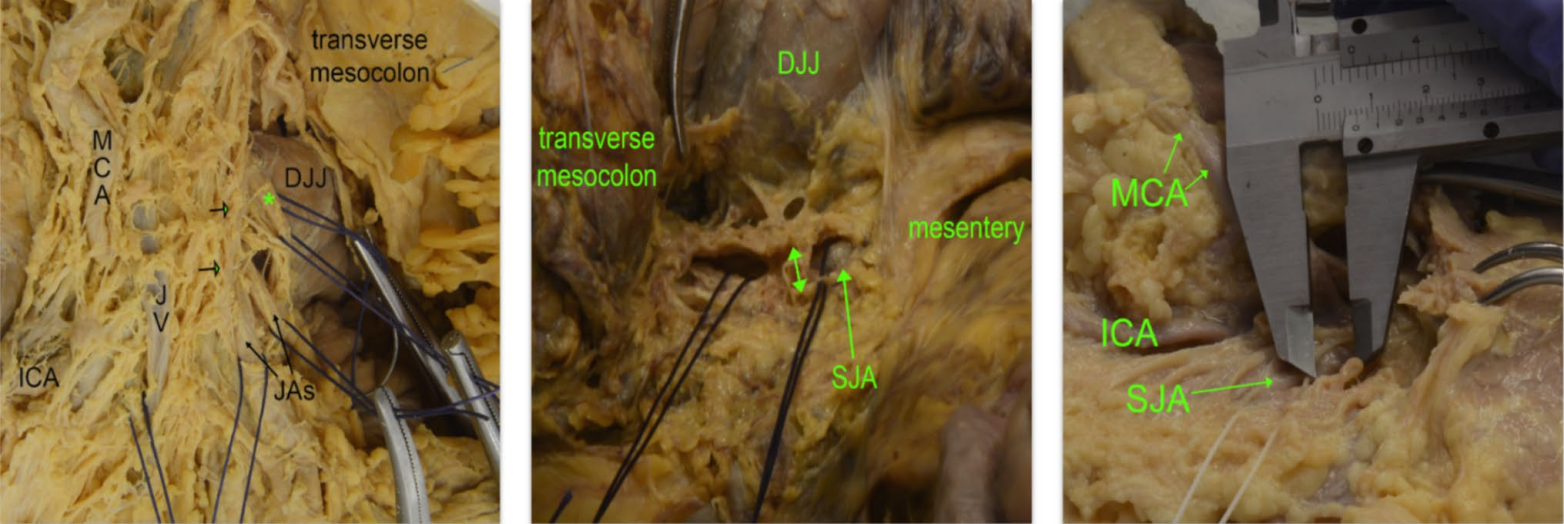
Anatomical dissection of the superior jejunal artery’s lymphovascular bundle. *ICA* Ileocolic artery; *MCA* middle colic artery; *JAs* jejunal arteries; *DJJ* duodenojejunal junction; *SJA* superior jejunal artery; green^*^SJA,^→^collector lymph vessel along the SMA left-hand side,^←→^SJA’s lymphatic clearance

Dissection of the specimens clearly revealed the posterior pancreatic fascia of Treitz, as well as the anterior pancreatic fascia of Fredet and the visceral fascia of Toldt), all of which are presented as thin yet distinct structures. [23, 24] When careful dissection of the lymphatics was done, the pattern of lymphatic drainage was clarified: a rich communication network of lymphatics was revealed within the proximal jejunal mesentery, i.e., between the lymphatics of the SJA and its neighboring JAs. On the other hand, no such lymphatic branching extended upwards into the transverse mesocolon lipolymphatic tissue. The lymphatic web following the proximal JAs showed a converging, conical pattern, with a narrowing of the lymphatic vessel web concentrating around the arterial origin from the SMA (Fig. 1). When measuring the distances of the cranial and caudal lymphatics following the SJA, the lymphatic clearances at the arterial origin were measured. The distances were 1.15 ± 0.53 mm and 0.6 ± 0.35 mm. Additionally, at a distance of 1 cm distally from the SJA’s origin (in a centrifugal direction), the clearances measured were 6.5 ± 2.3 mm and 2.5 ± 1.2 mm. In addition to the described lymphatic network, there are lymph vessels originating from the left colic angle and the transverse colon, which arch over the small bowel lymphatics in the form of a canopy, with a distinct connective tissue septum between them.

## Discussion

Surgical techniques for treating distal duodenal/proximal jejunal tumors remain vaguely defined. A standardized and personalized surgical approach tailored to the patient’s individual anatomy is required [25]. A key aspect of this is to ensure comparable numbers of harvested LNs (including central LNs of D3 volume) for operating surgeons/institutions in order to improve overall survival, as shown by Banipal et al [26]. Our research outlines several important considerations for surgeons dealing with duodenojejunal tumors. These include the position of the SJA origin, its lymphatic clearances, as well as the topography of the SJV.

Distal duodenal and proximal jejunal tumors are supplied through the SJA proper, IPDA, and their common trunk. There are significant variations in the vascular origins of the SJA. Specifically, the prevalence of the common IPDA–SJA trunk ranges from 25 to 71.4%.[27, 28]. The variability of the common trunk plays an important role in pancreatic surgery, where inadvertent vascular injury of the SJA proper can occur. According to Sakaguchi et al., the SJA proper originates from the left (58.4%) or dorsal (39.2%) side of the SMA, the remainder arising from the right [29]. Although the CVL attempts of the SJA have been reported in the literature, its retropancreatic origin has not been highlighted as a challenge [30, 31]. Our results indicate that a surgeon may encounter a retropancreatic SJA in 17.5% of patients. The anatomical dissections revealed a somewhat higher percentage (30%), which can be attributed to the smaller sample size. If the surgeon chooses D3 LN dissection (removal of all mesenteric fatty tissue surrounding the SMV and SMA from the level of the ICA to the tumor-feeding vessel), following the SMA within its vascular sheath allows identification of the SJA and its safe division. In addition, the SJA’s relation to the MCA origin and SV facilitates navigation. If a D2 procedure is intended, identifying the SJA can be more difficult. In cases where the origin of the SJA is retropancreatic, establishing a retropancreatic tunnel is essential to safely ligate the retropancreatic SJA. The positioning of the SJA in relation to the pancreatic notch, or the lower border of the pancreas, significantly impacts the surgical approach; a cranial variant poses a greater risk of damaging the gland. Consequently, this may extend the duration of surgical dissection and possibly result in postoperative complications. At times, the adjacent caudal JA is recognized as the tumor-feeding vessel. A semi-automated or manual 3D reconstruction of the blood vessels is of considerable help when clarifying the tumor-feeding artery’s position and its relation to other anatomical landmarks during surgery [32, 33].

**Fig. 2:**
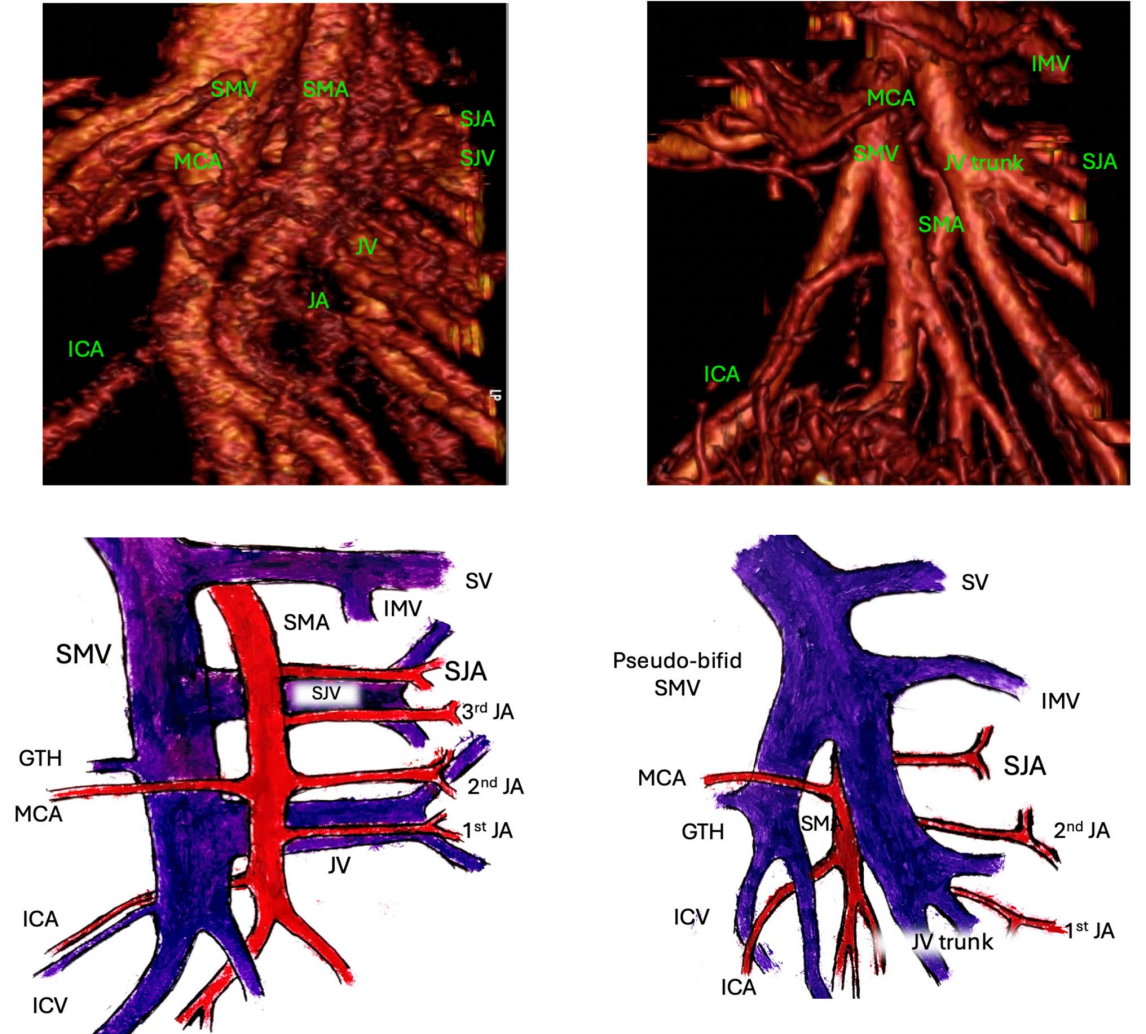
Three-dimensional SMA/SMV reconstructions and illustrations of the spatial relationship between the superior jejunal artery and the superior jejunal vein. On the left, the posterior SJV course, on the right, the anterior SJV course (pseudo-bifid SMV); *SMA* superior mesenteric artery; *SMV* superior mesenteric vein; *ICA* ileocolic artery; *MCA* middle colic artery; *GTH* gastrocolic trunk of Henle; *ICV* ileocolic vein; *JA* jejunal artery (i.e., 2nd JA); *SJA* superior jejunal artery; *SJV* superior jejunal vein; *JV* jejunal vein; *IMV* inferior mesenteric vein; *SV* splenic vein

Another challenge during the CVL of the tumor-feeding artery is the position of the SJV [34]. The SJV, a large-caliber tributary of the SMV, can cross the SMA anteriorly or posteriorly when approaching its confluence (Fig. 2). If the SJV obscures the origin of the SJA anteriorly (in 29.3% of cases), there is an increased risk of vascular injury and bleeding during ligation and division of the tumor-feeding vessel at its origin [35]. The left SMV trunk (pseudo-bifid SMV) and the IMV crossing the SMA anteriorly both contribute to the complexity. In such cases, it may be wise to place the SJV in a vessel loop in order to manipulate it when accessing the SJA origin; SJV ligation is rarely required. Current literature supports the notion that ligating the SJV is generally safe if additional jejunal venous trunks and the distal SMV are present and preserved [36]. However, no data on the position of this ligation have been reported. Literature states that thrombosis in large veins can occur when the ligature of a tributary is not performed at its confluence [37]. All of the above challenges seem to imply that the use of a 3D vascular anatomy reconstruction can be beneficial when operating on these patients, both to evade complications or to resolve them at surgery [38].

**Fig. 3:**
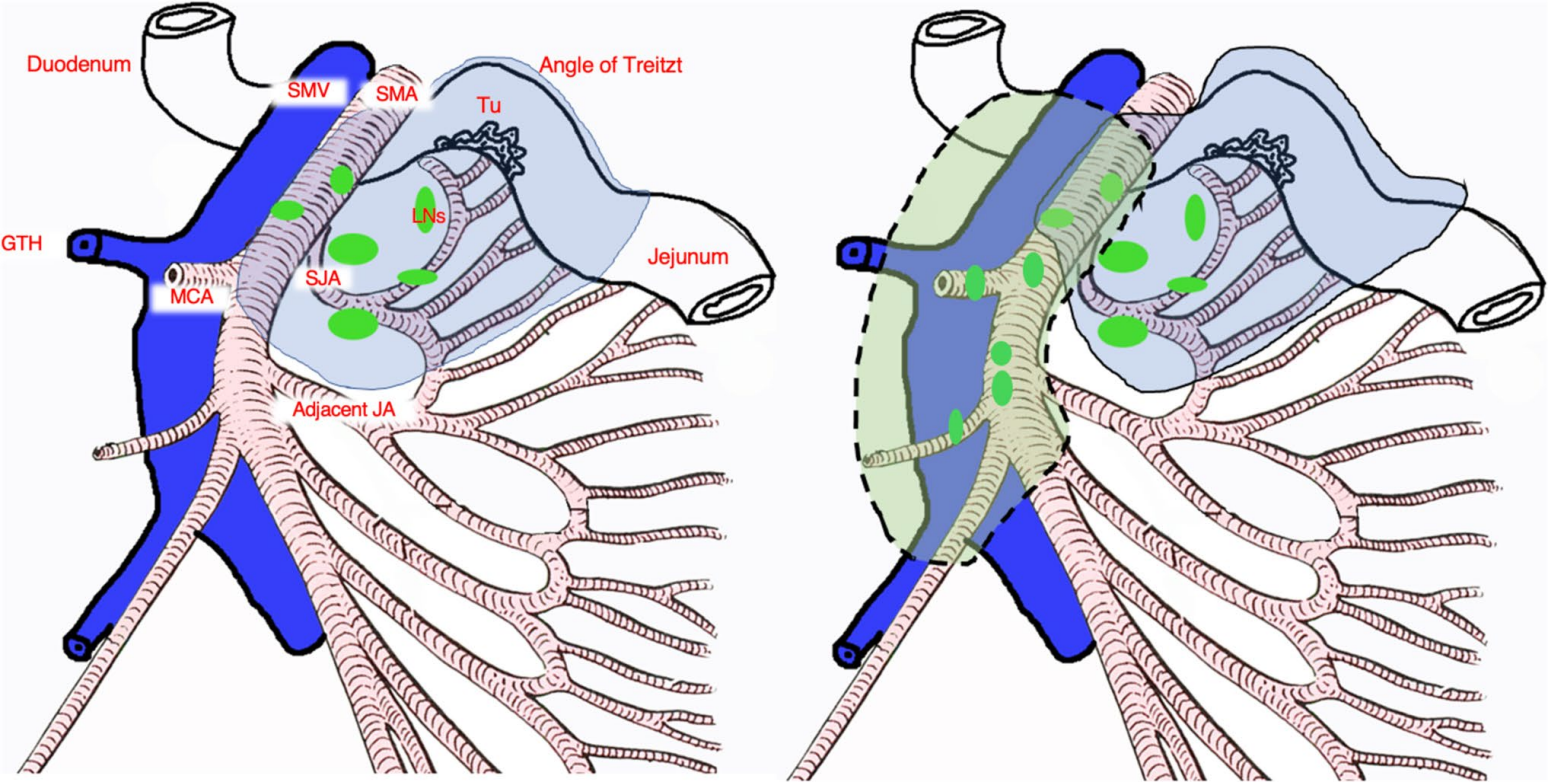
The proposed technique of the D2 and D3 mesenterectomy in radical small bowel resection for the distal duodenal and jejunal tumors. On the left, blue area: D2 mesenterectomy; on the right, green area bordered with dashed line: extended D3 mesenterectomy, blue area: D2 mesenterectomy; *SJA* superior jejunal artery; *JA* jejunal artery; *LNs* lymph nodes; *SMA/SMV* superior mesenteric artery/vein; *MCA* middle colic artery; *GTH* the gastrocolic trunk of Henle; *Tu* tumor

Recent dynamic contrast-enhanced studies examined the centripetal lymph flow from the bowel wall along the arteries toward the root of the mesentery [39, 40]. Compared to the well-studied colonic and mesopancreatic lymphatic pathways, the neighboring lymphatics associated with the duodenojejunal mesentery in SB resections are vaguely examined [41, 42]. This study defines the width of lymphovascular bundles based on lymphatic clearances observed during cadaver dissections. Our earlier research, which employed immunohistochemistry to verify lymph vessels histologically, reported no notable anatomical changes that would affect the shape or clearance of the lymphovascular bundles [13]. While JAs have both cranial and caudal arteries in their vicinity, the SJA is missing this proximal artery in all cases. This consequently makes its cranial lymphatic clearance somewhat wider when compared to the previously published findings on the jejunal and ileal lymphovascular bundles (1.15 ± 0.53 mm at SJA origin / 6.5 ± 2.3 mm at 1 cm distal to the origin) [43, 44]. In this manner, the lymphatic network following the SJA showed a converging, conical structure, with the lymphatic vessels narrowing around the origin of the artery. As reported in our earlier study, the caudal lymphatic clearance at the level of SJA origin is lower than that associated with the arteries of the right colon (2.8–6.3 mm) [13]. Nevertheless, the JAs are more prevalent and positioned closer together, leading to a denser lymphatic network [43]. A detailed network between lymphatics of the SJA and its adjacent JA has previously been discussed by Hollinshead in the context of tumor spreading at surgery [3]. In short, D2 dissection should include the removal of mesenteric fatty tissue around the SJA origin, including the specified cranial and caudal lymphatic clearances, as well as lymphadenectomy along the adjacent JA to the level of the first arcade. If extended D3 dissection is planned, the goal is to approach the SMA axis through caudal to cranial dissection in the D3 volume and remove the fatty mesenteric tissue containing the central LNs in the D3 volume, simultaneously identifying all JA origins, and thereafter continue with the D2 volume removal as previously described. When extended D3 mesenterectomy is performed, the resected specimen is removed en bloc with the D3 volume having the shape of an axe (Fig. 3).

This study’s strength lies in the detailed anatomical dissections conducted by anatomists MS and BVS. While the sample size for anatomical dissection was small, it seems sufficient for the conclusions made. However, a limitation is that LNs were not counted during the dissection, as the focus was primarily on the course of the lymph vessels. Additionally, the meticulous 3D reconstructions of the vascular anatomy provided by BVS and validated during surgery indicate an adequate sample size. The integration of these two complementary methods enhances the study’s strength by allowing data extrapolation to surgical techniques in the treatment of distal duodenal and proximal jejunal tumors.

## Conclusion

To achieve adequate lymphatic clearance, it is crucial to ligate the tumor-feeding SJA at its origin. The conical arrangement of the SJA lymphatics underscores the necessity of a mesenterectomy extending both cranially and caudally and including lymphadenectomy along the adjacent JA to the level of the first arcade. A retropancreatic SJA origin can present a challenge to the operating surgeon, especially if obscured by the SJV. The extrapolated results of this study support the proposed extended D3 mesenterectomy technique for treatment of duodenojejunal tumors.

## Supplementary Information

Below is the link to the electronic supplementary material.

Supplementary file1 (MOV 231662 KB)

## Data Availability

Available on reasonable request.
