# Correction: Lymphatic and vascular anatomy define surgical principles for radical treatment of distal duodenal and proximal jejunal tumors

**DOI:** 10.1007/s00464-025-11994-w

**Published:** 2025-07-23

**Authors:** Teodor Vasic, Milena Stimec, Bojan Vladimir Stimec, Bjørn Edwin, Dejan Ignjatovic

**Affiliations:** 1 Clinic for Digestive Surgery, Faculty of Medicine, University Clinical Centre of Serbia, University of Belgrade, Belgrade, Serbia; 2 Anatomy Unit, Faculty of Medicine, University of Geneva, Geneva, Switzerland; 3 Intervention Centre, Oslo University Hospital, Oslo, Norway; 4 Department of Digestive Surgery, Akershus University Hospital, Oslo, Norway; 5 Institute of Clinical Medicine, Faculty of Medicine, University of Oslo, Oslo, Norway

**Correction to: Surgical Endoscopy** 10.1007/s00464-025-11909-9

The original online version of this article was revised to reflect the presence of Electronic Supplemental Material. A link to this material has been added to the online version of the article.

The original article has been corrected.

